# Australia’s first transition to professional practice in primary care program for graduate registered nurses: a pilot study

**DOI:** 10.1186/s12912-017-0207-5

**Published:** 2017-03-23

**Authors:** Christina Aggar, Jacqueline Bloomfield, Tamsin H. Thomas, Christopher J. Gordon

**Affiliations:** 1 Health & Human Sciences, Southern Cross University, Queensland, Australia; 2 Sydney Nursing School, University of Sydney, Sydney, NSW 2006 Australia

**Keywords:** Primary health care, Primary care nursing, New graduate nurses, Transitional programs, General practice, Clinical competence

## Abstract

**Background:**

Increases in ageing, chronic illness and complex co-morbidities in the Australian population are adding pressure to the primary care nursing workforce. Initiatives to attract and retain nurses are needed to establish a sustainable and skilled future primary care nursing workforce. We implemented a transition to professional practice program in general practice settings for graduate nurses and evaluated graduate nurse competency, the graduate nurse experience and program satisfaction. This study aimed to determine whether a transition to professional practice program implemented in the general practice setting led to competent practice nurses in their first year post-graduation.

**Methods:**

A longitudinal, exploratory mixed-methods design was used to assess the pilot study. Data were collected at three times points (3, 6, 12 months) with complete data sets from graduate nurses (*n =* 4) and preceptors (*n =* 7). We assessed perceptions of the graduates’ nursing competency and confidence, satisfaction with the preceptor/graduate relationship, and experiences and satisfaction with the program. Graduate nurse competency was assessed using the National Competency Standards for Nurses in General Practice. Semi-structured interviews with participants at Time 3 sought information about barriers, enablers, and the perceived impact of the program.

**Results:**

Graduate nurses were found to be competent within their first year of clinical practice. Program perceptions from graduate nurses and preceptors were positive and the relationship between the graduate nurse and preceptor was key to this development.

**Conclusions:**

With appropriate support registered nurses can transition directly into primary care and are competent in their first year post-graduation. While wider implementation and research is needed, findings from this study demonstrate the potential value of transition to professional practice programs within primary care as a nursing workforce development strategy.

## Background

The increasing prevalence of chronic illness and multi-morbidity evident in the Australian population has considerable implications for the nursing workforce, particularly in primary care. Currently there is a nursing shortage in primary care, which is compounded by an ageing nursing workforce [1]. It is anticipated that this will continue to grow [2]. Providing a structured transition program for graduate nurses wanting to work in primary care is one avenue that has not been undertaken and evaluated to date in Australia. This paper reports a pilot study that was developed to support the transition of new graduate registered nurses (hereafter ‘graduate nurses’) to primary care.

Population ageing and the increasing prevalence of complex chronic disease and multiple co-morbidities in Australia is putting pressure on general practices. Between 2011 and 2012, chronic diseases accounted for 36% of all problems managed in general practice in Australia [3]. It is predicted that this number will continue to increase. International evidence demonstrates that nurses provide cost-effective, high-quality primary health care services to people with chronic and complex conditions [4]. In Australia, data confirms that practice nurses have a central role in the effective management and integration of care for people with chronic and complex health conditions and that these nurses are integral for health service provision [5]. At present, however, there is a considerable nursing shortage in primary care in Australia and it is predicted that this will be up to 110,000 nurses by 2025 [6]. Strategies to attract and retain new nurses in primary care settings are now needed in order to establish a sustainable and skilled nursing workforce, especially to meet future population health needs [7].

Currently, the majority of graduate nurses commence employment in acute-care settings, typically in transition to professional practice programs implemented predominantly within hospital settings [8]. These programs aim to promote nursing student to registered nurse transition through formal and informal education, facilitation of workplace learning, professional socialisation, preceptorship, and practice development, in a supportive environment. Importantly, it is also anticipated that by developing levels of competence and confidence commensurate with the professional role, graduate nurses participating in these programs will develop a commitment to a career in nursing, thereby impacting workforce retention [9, 10]. To date, graduate nurses have not had opportunities to transition directly into primary care settings in Australia, as these programs have not been established. As such, there is a need to create a suitable environment for primary care graduate nurse transition programs to facilitate growth of the primary care nursing workforce.

To address the shortage of nurses in primary care we developed and implemented a novel transition to professional practice program where registered nurses could transition directly from university into primary care settings. The structured program included preceptorship, education, and support, tailored specifically to primary care nursing needs. As this program was the first of its kind in Australia, it was imperative that it was piloted to determine graduate nurse competency, confidence, and views about program support. Herein, we present data from the graduate nurses and program nurse preceptors.

### Study objectives

The study sought to determine whether a transition to professional practice program implemented in the general practice setting led to competent practice nurses in their first year post-graduation. It had three main objectives:

1. to determine graduate nurse competency across the program;
2. to evaluate the graduate nurse experience during the program;
3. to evaluate graduate nurse and nurse preceptor program satisfaction.

### The program

The development of the Transition to Professional Practice in Primary Care Program for graduate nurses commenced in March 2014, in collaboration with the Northern Sydney Medicare Local (NSML). Medicare Locals, a component of the Australian Government National Health Reform, were a primary care initiative to improve coordination and integration of primary health care in local communities, address service gaps, and support people to navigate their local health care system [11]. Medicare Locals were replaced in 2015 by Primary Health Networks which have a similar remit [12]. An Advisory Committee consisting of national and local key stakeholders was established to provide guidance on the development and implementation of the program and met bimonthly. Committee members included representatives from NSML, the Australian Medicare Local Alliance (AMLA; the peak body supporting Medicare Locals), the Australian Primary Health Care Nurses Association (APNA), and the Royal Australian College of General Practitioners (RACGPs).

The 12-month program, which commenced in January 2015, comprised employment for a 12-month period in general practice settings, preceptor and educational support, overseen by a transition facilitator. The graduate nurses who participated in the program were employed in general practices within the NSML. It was anticipated that each graduate would rotate through two practices during the program to provide a wider scope of experiential learning [13]. The graduate nurses were provided with training resources, online educational materials, study days and networking opportunities. Additionally, graduate nurses were supported by preceptors who were registered nurses employed within the same general practice. The preceptors undertook a formal structured training program consisting of 8 h of face-to-face training and follow-up consultations in their workplace. The primary role of the preceptors was to provide ongoing support to the graduates during the transition year. The practical implementation of the program was overseen by a Transition Program Coordinator, who supported both the graduates and preceptors.

### Research design

A longitudinal, exploratory study consisting of a mixed-method design was used. In light of the exploratory nature of the study, the integration of quantitative and qualitative data, assisted in diminishing potential weaknesses of a single approach and enhanced validity of findings [14]. We employed this methodology as we wanted to explore thematic aspects of the transition program that became apparent with the qualitative component. In this way, we can provide recommendations for future programs and issues that may arise that were not ascertained from quantitative data alone.

## Methods

Graduate nurses and nurse preceptors completed structured questionnaires administered at three time-points: Time 1 (3 months into the first rotation), Time 2 (6 months - start of second rotation), and Time 3 (12 months - end program completion). Baseline data (Time 1) was collected at 3 months to allow the graduate nurses to become familiar with the practice setting and the skills required. Graduate nurses, preceptors and the program coordinator participated in semi-structured interviews at the completion of the program. General practitioners were invited to provide feedback but all declined this offer.

### Participant recruitment

The program was advertised nationally and the timing of the recruitment of the graduate nurses was conducted in parallel to other state and territory transition programs for graduate nurses. Graduate nurses were recruited via an advertisement posted on a national employment website. Over 100 applications were received, eighteen nurses were offered an interview and a total of eight nurses were offered a place in the program, of which six nurses accepted a position and were placed in general practices. Registered nurses from the participating general practices were identified as potential preceptors and all, but one, indicated that they would be willing to be involved. Some of these registered nurses specified that they had already provided preceptorship to new nursing staff at the practices; however, none had been involved with graduate nurses and none had formal preceptorship training to support this role.

### Data collection

Data collection instruments used in this study comprised full or abbreviated versions of previously validated questionnaires. At Time 1 graduate nurses and preceptors completed a demographics questionnaire, which collected information about the general practice where they worked such as the number of GPs and nurses employed, number of patients seen per day, and time spent with each patient. They also reported how many hours per week the graduate nurse and preceptor spent together. Graduates also completed questionnaires related to nursing competency, nursing experience and satisfaction with the graduate nurse’s preceptor and the program. These were administered at the three-month period (Time 1) to provide a baseline that could be used to compare longitudinal data during the program (6 and 12 months). At Time 2 graduate nurses completed the same questionnaires about nursing competency, nursing experience, program support and preceptor satisfaction and this was also repeated at time 3. In addition, the graduate nurses were asked if their experience of the program would affect their future career decisions.

Preceptors completed questionnaires about how they perceived the graduate nurse’s competence; their expectations and experiences of the program including their relationship with the graduate nurse; and their perceptions of the benefits and rewards of preceptorship, and commitment to clinical supervision. At Time 3 the graduate nurses’ competence was assessed by preceptors using the National Practice Standards for Nurses in General Practice [15]. They relate to best practice for nursing work in the general practice setting. The preceptors scored the performance indicators against the standard that the graduate nurse had achieved. The 22 Practice Standards are categorised in four domains relevant to nursing in general practice namely: Professional Practice, Nursing Care, General Practice Environment and Collaborative Practice [15]. Examples of Standards contained within each domain are presented in Table 1.

**Table 1:** Examples of the 22 National Practice Standards for Nurses in General Practice within four domains [15]

Domain	Example standards
Professional practice	Standard 1: Demonstrates an understanding of primary health care principles and nursing in general practice. Standard 2: Provides nursing care consistent with current nursing and general practice standards, guidelines, regulations and legislation.
Nursing care	Standard 6: Demonstrates the knowledge and skills to provide safe, effective and evidence-based nursing care. Standard 10: Understands diversity in the Practice community and facilitates a safe, respectful and inclusive environment.
General practice environment	Standard 13: Demonstrates proficiency in the use of information technology, clinical software and decision support tools to underpin health care delivery. Standard 16: Contributes to quality improvement and research activities to monitor and improve the standard of care provided in general practice.
Collaborative practice	Standard 20: Builds and maintains professional and therapeutic relationships with consumers, their families and/or support person(s). Standard 22: Liaises effectively with relevant agencies and health professionals to facilitate access to services and continuity of care.

Semi-structured interviews with all participants (graduate nurses, preceptors, and the program coordinator) were conducted at the end of the program (12 months). These explored what they perceived was the impact (including benefits and negatives) of the program for all parties including the graduate nurse, their preceptor, the GPs, and the patients. Participants were also asked about perceived barriers to the implementation of the program and how these might be overcome.

### Measures

There is a lack of primary-care nursing questionnaires related to graduate nurse programs and particularly participant experiences during the program. As such, we needed to use questionnaires that measured constructs of relevance to the study. In addition, we modified some questionnaires to provide primary care context whilst maintaining the integrity of the tools' validity. Five instruments were used to collect data from graduates and preceptors during the study. Tools included:

1) Six Dimension Scale of Nursing Performance [16] which assessed the graduate nurse and preceptors’ perceptions of the graduate’s nursing competency; 2) Casey-Fink Graduate Nurse Experience Survey [17] which assessed the graduate’s perceived confidence; 3) Preceptor/Preceptee Satisfaction Questionnaire [18] which assessed graduates and preceptors' perceived satisfaction with the preceptor/graduate relationship in terms of preceptor behaviours; 4) Halfer-Graf Job/Work Environment [19] which assessed graduate's satisfaction with the program; and 5) Nurse Entry to Practice Program Evaluation [20] which assessed graduate nurses experiences of the program including rotations and their preceptor. A summary of these tools including alterations for use in this study, and when and to whom tools were administered is presented in Table 2.

**Table 2:** Data collection instrument summary including scoring and time points administered

Scale	Domains	Sample question and responding	Scoring and interpretation	Items used and excluded	Time points
Six Dimension Scale of Nursing Performance; [16]	Graduate competence. Subscales: 1. Leadership – guide others, accept responsibility; 2. Teaching and Collaboration – teach family using available resources; 3. Planning and Evaluation – identify patient needs, develop care plan; 4. Interpersonal Relations and Communication – build trust, teach patients, ask for help.	*“Help a patient communicate with others”.* Two 4-point Likert scales. 1. Frequency of task: *Not Expected in this Job – Frequently* 2. Skill: *Not Very Well – Very Well*	Total score and subscales: Mean skill score for items with frequency score >1. Higher scores indicate greater competence.	31 items. 12 irrelevant items excluded e.g. critical care subscale (specific to hospital settings).	*GN time 1,2,3* *PN time 1,2,3*
	Graduate professional development. Ability to display self-direction, and use opportunities for growth and development.	*“How well do you accept and use constructive criticism”.* 4-point Likert scale. Skill: *Not Very Well – Very Well*	Total score: mean skill score. Higher scores indicate greater competence.		
Casey-Fink Graduate Nurse Experience Survey; [17]	Graduate confidence. Subscales: 1. Support, encouragement, and feedback; 2. Organising/prioritising patient needs and workload; 3. Communication/leadership including making suggestions regarding patient care; 4. Professional satisfaction satisfying, exciting, challenging work with opportunities for practice, and role models.	*“I feel confident communicating with general practitioners”.* 5-point Likert scale. *Strongly Disagree – Strongly Agree*	Total score: summed (prorated). Subscales: mean. Higher scores indicate greater confidence.	22 items. 2 irrelevant items excluded: i.e. helping dying patients and delegating to the nursing assistant.	*GN time 1,2,3*
New Graduate Program Satisfaction; [19]	Satisfaction with program. Perceived ability, as a result of the program, to develop work relationships, use resources effectively, ask questions, and contribute professionally.	*“The program has allowed me to have professional contributions valued”.* 4-point Likert scale. *Strongly Disagree – Strongly Agree*	Total score: summed (prorated). Higher scores indicate greater satisfaction with program.	11 items. 10 irrelevant items excluded due to overlap with other tools i.e. confidence, leadership.	*GN time 1,2,3*
Nurse Entry to Practice Program Evaluation; [20]	Opportunities provided by the program including access to a knowledgeable/helpful preceptor, timely feedback, and opportunities to learn and develop.	*“The program offers me the opportunity to develop professionally as a nurse”.* 5-point Likert scale. *Strongly Disagree – Strongly Agree*	Total score: mean. Higher scores indicate more perceived opportunities provided.	9 items. Nil excluded.	*GN time 1,2,3* *PN time 1,2,3*
Preceptor/Preceptee Satisfaction Questionnaire; [18]	Satisfaction with relationship between preceptor and preceptee.	*“My preceptor welcomes my questions”.* 5-point Likert scale. *Strongly Disagree – Strongly Agree*	Total score: summed (prorated). Higher scores indicate greater satisfaction with relationship.	19 items. 2 items excluded from GN survey only (preceptor specific questions).	*GN time 1,2,3* *PN time 1,2,3*

*Abbreviations*: *GN* graduate nurse, *PN* practice nurse (preceptor)

### Data analysis

Within and between-group analysis was conducted and significant differences considered when *p <* 0.05. Within-group differences across time were analysed using Student paired *t*-tests or Wilcoxon signed-rank tests for non-parametric data. Between-group differences were analysed using Student independent *t*-tests or Mann Whitney U tests for non-parametric data. As the program was a pilot, the study was exploratory in nature and the probability of Type I errors arising due to multiple comparisons was not considered a major concern. As such, we did not apply Bonferroni corrections for multiple comparisons as the technique can be overly conservative and may exclude potentially important results which have clinical translation implications. Quantitative data were analysed using SPSS 22 (IBM); computation of scores for each scale is outlined in Table 2. Qualitative data was subjected to thematic analysis using Nvivo; data were assessed for themes independently by three researchers who then discussed their findings.

## Results

Six graduate nurses commenced the program and four completed the whole year. One graduate nurse withdrew to commence a position in a large inner-city general practice and the other graduate nurse withdrew due to family relocating overseas. Six general practices were included at the start of the program and four were involved after the year; two practices withdrew as a result of the withdrawal of the graduate nurses. Nine nurse preceptors commenced the program and 2 were excluded as the graduate nurse for whom they were a preceptor left the program. General practices were large, with an average of 8 GPs and 3 RNs; graduates saw an average of 20 patients per day. Graduates saw their preceptors between 1 and 4 days per week for 1—7 h per day; further details can be seen in Table 3.

**Table 3:** General practice demographics including contact hours between graduates and preceptors

General practice	Total GPs	Total RNs	Patients per day (graduate)	Time with patient (mins)	Days per week with preceptor	Hours per day with preceptor
1	10	3	16	22	0.5	4
2	7	3	30	10	4	1
3	8	3	20	15	4	7
4	8	2	13	15	3	5
Mean	8	3	20	16	3	4

*Abbreviations*: *GPs* general practitioners, *RNs* registered nurses

The total number of participants who returned surveys at all three time points included four graduate nurses and seven preceptors. This small, mixed-methods pilot study was largely exploratory and the small sample size (4 graduates and 7 preceptors) did not provide sufficient power to detect significant changes. Therefore, we mainly report descriptive level data only in this pilot study as statistical significant differences were not deemed highly conclusive due to the low sample size. As such quantitative data was used primarily to elucidate trends which could be further explored in qualitative, semi-structured interviews. Semi-structured interviews were conducted with four graduate nurses and seven nurse preceptors. Herein, we report the findings from the quantitative analysis and support this with qualitative responses. These are presented in a sequential manner for ease of interpretation.

### Graduate nurse competency

Graduates competency was evaluated according to the National Practice Standards for Nurses in General Practice [15]. These practice standards relate to professional practice, nursing care, general practice environment and collaborative practice [15]. The graduate nurses were assessed as competent by their preceptor. Not all performance indicators were completed as this was not realistic in the one year of practice; however, the graduate nurses attained the majority of indicators demonstrating their competency.

The Six-D Scale [16] was used to assess perceived performance. Whilst there was a decrease in overall perceived nursing performance between Time 1 (*M =* 3.2) and Time 2 (*M =* 2.9), at the end of the program, graduates’ perceptions of their own performance had increased (*M =* 3.3, all *p >* 0.05); Table 4. Graduate nurses exhibited increases in leadership (Time 1 *M =* 3.6, Time 3 *M =* 3.8), planning (Time 1 *M =* 2.9, Time 3 *M =* 3), and professional development (Time 1 *M =* 3.3, Time 3 *M =* 3.4) subscales. Although these increases were not statistically significant, there was a global increase from baseline.

**Table 4:** New graduate perception of their own nursing performance (Six dimension scale of nursing performance [16])

	Time 1 (*n =* 4)		Time 2 (*n =* 4)		Time 3 (*n =* 4)		*p* value
	Range	Mean (SD)	Range	Mean (SD)	Range	Mean (SD)	NS
Six dimension total	2.7–3.7	3.2 (0.49)	2.3–3.6	2.9 (0.6)	2.7–3.6	3.3 (0.42)	NS
Leadership subscale	3–4	3.6 (0.51)	2–4	3 (0.98)	3–4	3.8 (0.5)	NS
Teaching subscale	2.3–3.4	3 (0.5)	1.4–3.2	2.4 (0.73)	2.4–3.1	2.8 (0.33)	NS
Planning subscale	1.8–3.5	2.9 (0.72)	2–3.3	2.7 (0.58)*	2.3–3.5	3 (0.59)*	NS
Communications subscale	3.3–4	3.7 (0.41)	2.8–3.9	3.3 (0.51)	3.1–4	3.7 (0.43)	NS
Professional development subscale	2.7–3.9	3.3 (0.65)	2.6–3.9	3.4 (0.63)	2.8–4	3.4 (0.68)	NS

*NS* non-significant (*p >* 0.05)

*Within-group change over time. Wilcoxon signed-rank; *Z* = 1.84, *p* = 0.066

The qualitative response below typified the impact of the planning and communication competency subscales during the program:

*“… multidisciplinary communication, working in a team very closely, so that’s something that you probably don’t so much get in a hospital, I feel comfortable to go and knock on a doctor’s door and ask [them] a question” (Graduate 1; G1)*

Preceptors also rated the graduate’s performance using the Six-D Scale [16]. The preceptors tended to rate the performance of the graduates lower at Time 3 (*M =* 3.2) compared to the graduates self-rating (*M =* 3.3, *p* = 0.70); Table 5. Nevertheless, compared to the graduates’ self-perception, preceptors regarded the graduates had better teaching (graduate *M =* 2.8, preceptor *M =* 2.9) and planning (graduate *M =* 3, preceptor *M =* 3.4) competency at the end of the graduate program (all *p >* 0.05). Interestingly, the preceptors scored the graduates’ overall performance higher at Time 1 and 2 compared to the graduate, suggesting that graduates’ self-appraisal may have been under-represented earlier in the program. A preceptor said:

*“… we trained her up and everything. And she quickly became very competent. So there was nothing she couldn’t do. She also taught us a lot, she helped us a lot with the IT or the computers…” (Preceptor)*

**Table 5:** Preceptor perceptions of the new graduate’s nursing performance (Six Dimension Scale of Nursing Performance [16])

	Time 1 (*n =* 7)		Time 2 (*n =* 7)		Time 3 (*n =* 7)	
	Range	Mean (SD)	Range	Mean (SD)	Range	Mean (SD)
Six Dimension total	2.7–4	3.5 (0.49)	2.9–4	3.4 (0.41)	2.2–3.9	3.2 (0.61)
Leadership subscale	3–4	3.8 (0.43)*^1,2^	2–4	3.1 (0.64)*^1^	2–4	3.2 (0.72)*^2^
Teaching subscale	1.8–4	3.2 (0.74)	2.4–4	3.2 (0.47)	1.7–3.9	2.9 (0.86)
Planning subscale	1.8–4	3.4 (0.8)	2.5–4	3.3 (0.55)	2.5–3.8	3.4 (0.49)
Communications subscale	3–4	3.8 (0.39)*^3^	3–4	3.6 (0.43)*^4^	2–3.9	3.3 (0.67)*^3,4^
Professional development subscale	3–4	3.8 (0.36)*^5^	2.8–4	3.6 (0.51)*^5^	2–4	3.2 (0.72)*^5^

*Within-group change over time. Matching superscript indicates difference between scores

*^1^Wilcoxon signed-rank; *Z* = 2.04, *p =* 0.041

*^2^Wilcoxon signed-rank; *Z* = 2.03, *p =* 0.042

*^3^Paired-samples *t*-test; *t*(6) = 2.84, *p =* 0.03

*^4^Wilcoxon signed-rank; *Z* = 2.03, *p =* 0.042

*^5^Wilcoxon signed-rank; *Z* > 1.77 *p <* 0.076

### Graduate nurse experience

Graduate nurse experiences were assessed using the Casey-Fink Graduate Nurse Experience Survey [17]. Overall, graduate nurses’ confidence declined slightly from Time 1 (*M =* 73.3) to Time 2 (*M =* 68.5, *p* = 0.26), and appeared to remain constant from Time 2 to Time 3 (*M =* 68.7); Table 6. There were a large range of scores suggesting nursing graduates experienced quite different levels of confidence. For example, one graduate nurse said:

*“I just graduated so I need some improvement with my clinical skills so I can improve my confidence as well” (G2)*

Whereas another graduate nurse commented:

*“I was told… I wasn’t making very many mistakes; I was quite confident which came across in my practice” (G3)*

**Table 6:** Graduate nursing experience across the program year (Casey-Fink Graduate Nurse Experience Survey [17])

	Time 1 (*n =* 4)		Time 2 (*n =* 4)		Time 3 (*n =* 4)	
	Range	Mean (SD)	Range	Mean (SD)	Range	Mean (SD)
Confidence total	59.2–84	73.3 (12.26)	61.1–80.2	68.5 (8.62)	60.1–77.3	68.7 (8.89)
Support	2.9–4	3.5 (0.58)	3–4	3.4 (0.44)	2.9–3.9	3.3 (0.47)
Organising/prioritising	2.4–4	3.6 (0.8)	3–4	3.3 (0.48)	2.8–4	3.4 (0.59)
Communication/leadership	2.8–4	3.6 (0.59)	2.8–4	3.3 (0.55)	3–4	3.4 (0.52)
Professional satisfaction	1.3–4	3.1 (1.2)	2–3.7	2.8 (0.69)	1.7–3.7	2.7 (0.86)

The decline in average confidence towards the end of the program may have been influenced by the graduate nurses actively applying for post program nursing positions at the time of assessment and thus comparing their current skill set and confidence to starting a new, different job the following year. For example, one graduate nurse said:

*“I am confident and I have had the ability to grow my confidence here in general practice nursing [but]… it is going to be a whole different ball game in the hospital and I’m going to have to learn and gain my confidence all over again in that setting, get to know the people, get to know the environment, get to know how to do things all over again, because it is totally different… I would probably go on my first day confident because I have come out of here confident but then I’ll probably go straight back to square one and have to start again” (G1)*

Graduate nurse average scores for the *Support (range 3.3—3.5), Organising (range 3.3—3.6), and Communication (range 3.3—3.6)* subscales of the Casey-Fink Graduate Nurse Experience Survey were generally consistent between scales and over the course of the study. Interestingly, in relation to organisation/prioritisation, the graduates reported experiencing different levels of responsibility across the general practices. For example:

*“some practice nurses do a really broad variety of really amazing clinical things and given a lot more responsibility and then there’s other practices where your job description is very narrow and you’re not really given a lot of autonomy or responsibility and they’re the [practices] that you’re not going to get much out of. So it’s about finding practices to come on board with the program” (G3)*

The *Professional satisfaction* subscale of the Casey-Fink Graduate Nurse Experience Survey (Table 6) was lower than other subscales and continued to decline over the duration of the study (Time 1 *M =* 3.1, Time 3 *M =* 2.7) indicating that over time participants had a lower perception of the work being satisfying, exciting, and challenging*.* However, the graduate nurse interview responses were generally positive:

*“I think that some people that go into the hospital system after their first year are so burnt out by it and so shocked by how crazy it is that they think ‘what have I done’. I don’t feel like that at all. I feel like ‘wow, there are so many different avenues I can go into with nursing and this is just the beginning’… I think it’s really given me that sense” (G3)*

Some of the graduates suggested that, compared to the public health sector, opportunities for professional advancement in general practice are limited.

*“There’s a lot more career progression I think if you work for NSW Health rather than private practice” (G3)*

### Graduate primary care program satisfaction and evaluation

The program satisfaction and evaluation were assessed using the Halfer-Graf Job/Work Environment [19] and Nurse Entry to Practice Program Evaluation [20], respectively. Graduate nurses’ program satisfaction was consistently high over the study duration (range 3.3—3.4); Table 7.

*“It has been a very good experience for the whole year and we’ve been given a lot of education and support. I made a lot of friends, all the new grads, that’s been really good” (G2)*

**Table 7:** Graduate program satisfaction (New graduate nurse program evaluation [19])

	Time 1 (*n =* 4)		Time 2 (*n =* 4)		Time 3 (*n =* 4)		*p* value
	Range	Mean (SD)	Range	Mean (SD)	Range	Mean (SD)	
Program satisfaction total	2.1–4	3.3 (0.89)	2.3–3.9	3.3 (0.77)	2.5–4	3.4 (0.66)	NS

Similarly, the graduate nurses evaluated the overall opportunities provided by the program constructively. These included opportunities to ‘develop knowledge and practice skills’, and ‘develop professionally’. However, compared to Time 1 (*M =* 3.2) the graduates felt they had significantly less opportunities towards the end of the program (*M =* 3.1, *p < 0.05*); Table 8. This decrease in perceived opportunities may have been associated with changes in the nature of preceptorship as the graduates progressed through the year. This measure included the opportunity for ‘access to a designated preceptor for an agreed period of time’. As graduates progressed through the program, and gained experience, the required level of supervised preceptorship declined; however, it appeared graduates were comfortable with this decline. For example, one graduate nurse said:

*“the first practice I was in for the first 6 months was with 4 nurses and it really wasn’t a problem, I was never alone… and in my second practice I was being left alone a lot… I quite liked it because I don’t mind working alone and I still feel supported by the doctors. I don’t ever feel like there is no one I can ask” (G3)*

Another graduate nurse said

*“[I work] independently but there are always other nurses around… I love it, it’s more independent” (G4)*

**Table 8:** Graduate perceived opportunities provided (Nurse entry to practice program evaluation [20])

	Time 1 (*n =* 4)		Time 2 (*n =* 4)		Time 3 (*n =* 4)		*p* value
	Range	Mean (SD)	Range	Mean (SD)	Range	Mean (SD)	
Opportunities provided total	2.6–4	3.2 (0.6)	3.2–4	3.7 (0.35)^*^	2.8–3.4	3.1 (0.28)^*^	<0.05

*NS* non-significant

^*^Within-group change over time. Paired-samples *t*-test; *t*(3) = 3.6, *p =* 0.037

Additionally, the decrease in perceived opportunities provided over the course of the study may have been associated with graduates actively seeking employment towards the end of the study in hospital settings and other areas of primary care. This may have affected items reflecting the transferability of skills to other settings, for opportunities to ‘meet [their] learning needs’ or ‘develop professionally’. For example, one graduate nurse said

*“I’ve just started to get some interviews in hospitals which is good because I wasn’t for a while… I think they don’t realise how much actually goes on here that is translatable to hospital work” (G1)*

### Primary care preceptor/preceptee relationship satisfaction

The relationship between the graduate nurse and preceptor was evaluated using the Preceptor/Preceptee Satisfaction Questionnaire [18]. Graduate nurses generally rated the graduate/preceptor relationship highly (range 76.7—87.5), and reported having a preceptor who was available, encouraging, supportive, open, willing to share knowledge, and with whom they felt comfortable (Table 9). The average scores were above 75% satisfaction across all study time points. Interviews with graduate nurses reflected this satisfaction and provided support for practice nurses acting as preceptors. For example, one graduate nurse said:

*“the senior nurse took her role as preceptor very seriously, took a very structured approach to it, was always willing to teach me things, never expected me to work outside my scope of practice and never expected anything from me that I wasn’t ready to do, so I felt very well supported. And I felt like I could always ask if I didn’t know something, I never felt like any question was too stupid, and that’s the kind of learning environment you really need straight out of university because otherwise you’ll sweep your lack of knowledge under the carpet and you’ll get further behind. So that support really ensured that I learned as much as I possibly could about the job. She always had a good rational and evidence to back up why she was doing what she was doing and it was always current” (G3)*

**Table 9:** Graduate nurse ratings of preceptor satisfaction (Preceptor/preceptee satisfaction questionnaire [18])

	Time 1 (*n =* 4)		Time 2 (*n =* 4)		Time 3 (*n =* 4)		*p* value
	Range	Mean (SD)	Range	Mean (SD)	Range	Mean (SD)	
Preceptor satisfaction total	69–95	87.5 (12.48)	72–93	81 (9.49)	65–93	76.7 (11.82)	NS

The relationship between graduate nurses and preceptors varied between graduates. While the above example reflected a professional relationship, another graduate nurse had a different, but equally satisfactory, relationship with her preceptor and reported:

*“I feel very close to her. We’re just like friends and we help each other, support each other…. We’re just like friends. She’s very good, at teaching us, and also the emotional [support], and also like helping me to think about future career development which is very good” (G2)*

The preceptors rated their satisfaction with the graduate/preceptor relationship higher than the graduates, reporting 90% and higher average satisfaction (Table 10). Higher scores indicated greater agreement with the preceptor being available, encouraging, supportive, open, and willing to share knowledge.

**Table 10:** Preceptor ratings of preceptor satisfaction (Preceptor/Preceptee Satisfaction Questionnaire [18])

	Time 1 (*n =* 7)		Time 2 (*n =* 7)		Time 3 (*n =* 7)		*p* value
	Range	Mean (SD)	Range	Mean (SD)	Range	Mean (SD)	
Preceptor Satisfaction Total	94–100	97.9 (2.41)^*^	78–100	89.9 (8.8)^*^	81–100	91.9 (8.75)	NS*

*NS* non-significant

^*^Within-group change over time. Paired-samples *t*-test; t(6) = 2.34, *p =* 0.058

## Discussion

This pilot study sought to determine whether a transition to professional practice program implemented in the general practice setting led to competent practice nurses in their first year post-graduation. Although the generalisability of findings are limited by the small sample size, they demonstrate that transition to professional practice programs are transferable to primary care and support the clinical and professional development of graduate nurses. This is the first Australian transition to professional practice program implemented and evaluated in general practice, and whilst there was a small number of graduate nurses, the findings provide valuable insights for future program development.

The primary finding demonstrated clearly that graduate nurses who participated in the study were deemed competent in the general practice setting by the completion of the 12-month program. This was confirmed using the validated national standards [15] and nursing performance scale [16]. Further, the evidence from this study supports the applicability of the transition to professional practice program that supported these graduate nurses during their first year of nursing practice. The program was designed specifically for primary care nursing and requirements of first year graduates. Perceptions of the program by the graduate nurses and their preceptors were positive, indicating that they considered the level of educational and transitional support provided was appropriate. Although their experiences in general practice were generally reported as positive by the graduate nurses, the types of experiences varied. Opportunities to develop specific skills and knowledge was contingent on the type of practice the nurse was allocated and the demography of the patient population. While some graduate nurses were exposed to a diverse range of clinical situations requiring different skills and knowledge, others felt unchallenged. This has implications for job satisfaction, which is an important component of the new graduate experience and has been shown to be a strong predictor of intention to leave [21, 22]. Careful recruitment of general practices offering a wide-range of clinical experiences and the planning of rotations to maximise the diversity of learning opportunities and experiences for graduate nurses must be considered essential for future programs.

Importantly, study findings demonstrated the high satisfaction that practice nurses had with their relationship as preceptors to the graduate nurses. It is recognised that preceptors and/or mentors play a valuable role in the support of graduate nurses as they make the transition from the nursing student role [23, 24]. The preceptor role encompasses the provision of support as well as assisting the graduate to identify specific learning needs and opportunities where these can be met and can assist in developing the graduate’s skills, knowledge, confidence and clinical competence [23]. Findings from the study indicate that the preceptors effectively fulfilled this role and that this was valued by the graduates.

Preceptorship can also be viewed as a valuable professional development opportunity for practice nurses. Findings from a survey undertaken by Halcomb et al. [25] to identify the educational and professional development needs of practice nurses in New South Wales, Australia, highlighted their desire to engage in education and training, however, few opportunities for this currently exist. From a professional developmental stance, being a preceptor or mentor can enhance self-awareness, confidence, and leadership ability as well as the development of teaching and assessment skills [24]. Study findings indicated that the practice nurses were willing to undertake this role and that they viewed their preceptorship experiences as positive. This has implications for future and potentially wider implementation of the Transition to Professional Practice in Primary Care Program. In particular, access to formalised preceptor training with appropriate financial and leave support provided by employers must be considered if practice nurses are expected to fulfil this role. Furthermore, formal recognition of the role of practice nurse as preceptors may assist in the development of career structures, thereby positively impacting recruitment and retention.

Notably this study also provides a contrary view to the widespread belief in Australia that it is essential for new graduate nurses to begin their career in the acute sector to obtain the requisite knowledge, skills and experience to work competently in the general practice setting. These results show that graduate nurses can transition directly into primary care with a professional support program. As such, programs such as these would address the shortage of nurses in primary care and provide support for wider implementation of such programs as valuable nursing workforce development initiatives.

## Conclusion

In conclusion, this pilot study establishes that transition to professional practice programs can be implemented in Australian primary care settings. This is an important new finding for Australian contexts but has implications in other countries without programs to support nurses working in primary care. The transition to professional practice was shown to support the needs of the graduate nurses in their first year of practice and the preceptor-support model implemented lead to strong relationships which facilitated learning in this environment.

## Abbreviations

AMLA: Australian Medicare Local Alliance
APNA: Australian Primary Health Care Nurses Association
G1: Graduate 1
GP: General Practitioner
NSML: Northern Sydney Medicare Local
RACGPs: Royal Australian College of General Practitioners
